# Supplementary figures and images for: Characterization of 3D organotypic epithelial tissues reveals tonsil-specific differences in tonic interferon signaling
Source: PLoS One. 2023 Oct 4;18(10):e0292368. doi: 10.1371/journal.pone.0292368 (PMC10550192; doi:10.1371/journal.pone.0292368)

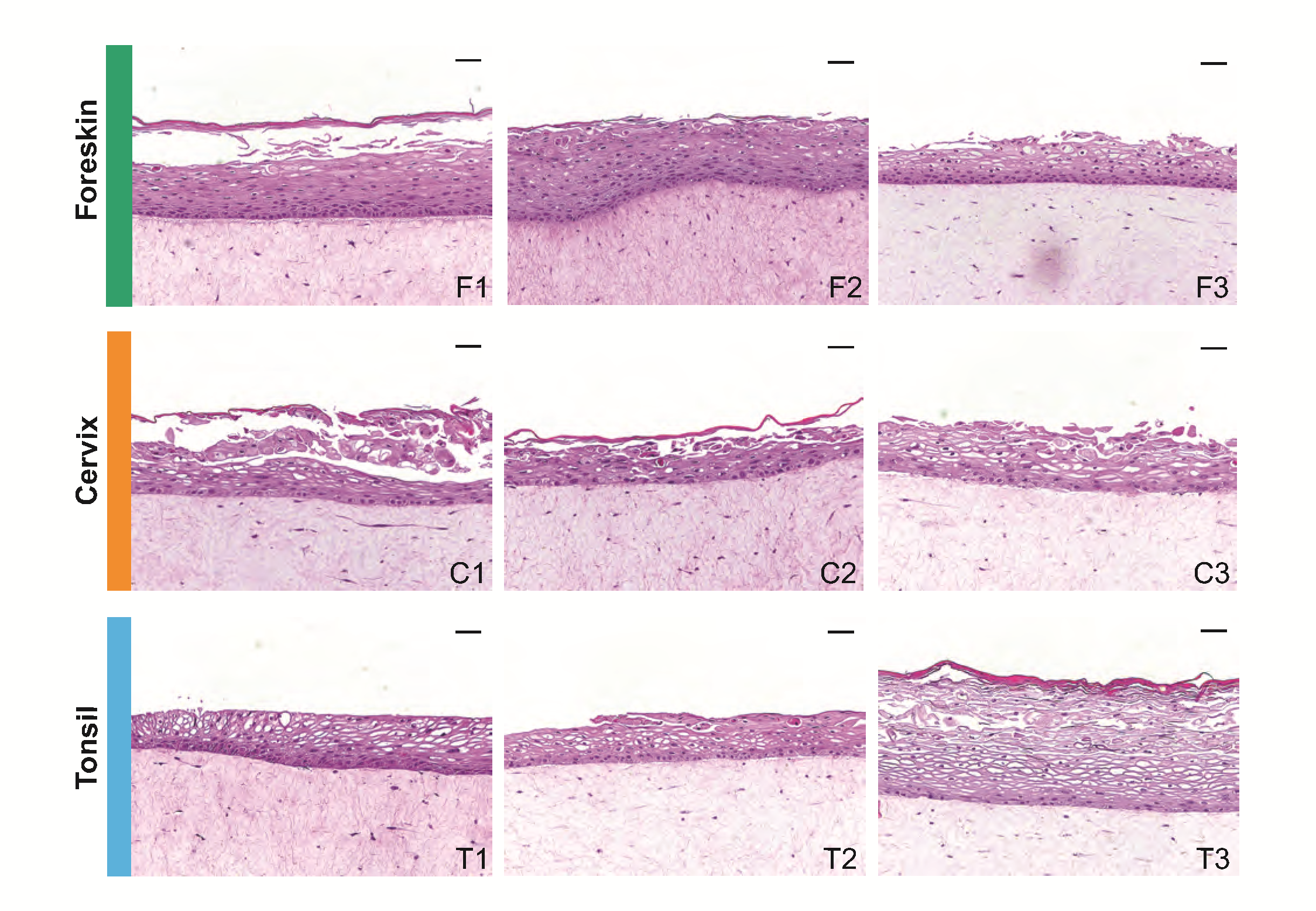

Supplement: S1 Fig — H&E micrographs of all nine independent donor 3D organotypic epithelial raft cultures (n = 3 for each tissue origin). Scale bars represent 50 μm. (TIF) [file pone.0292368.s001.tif]

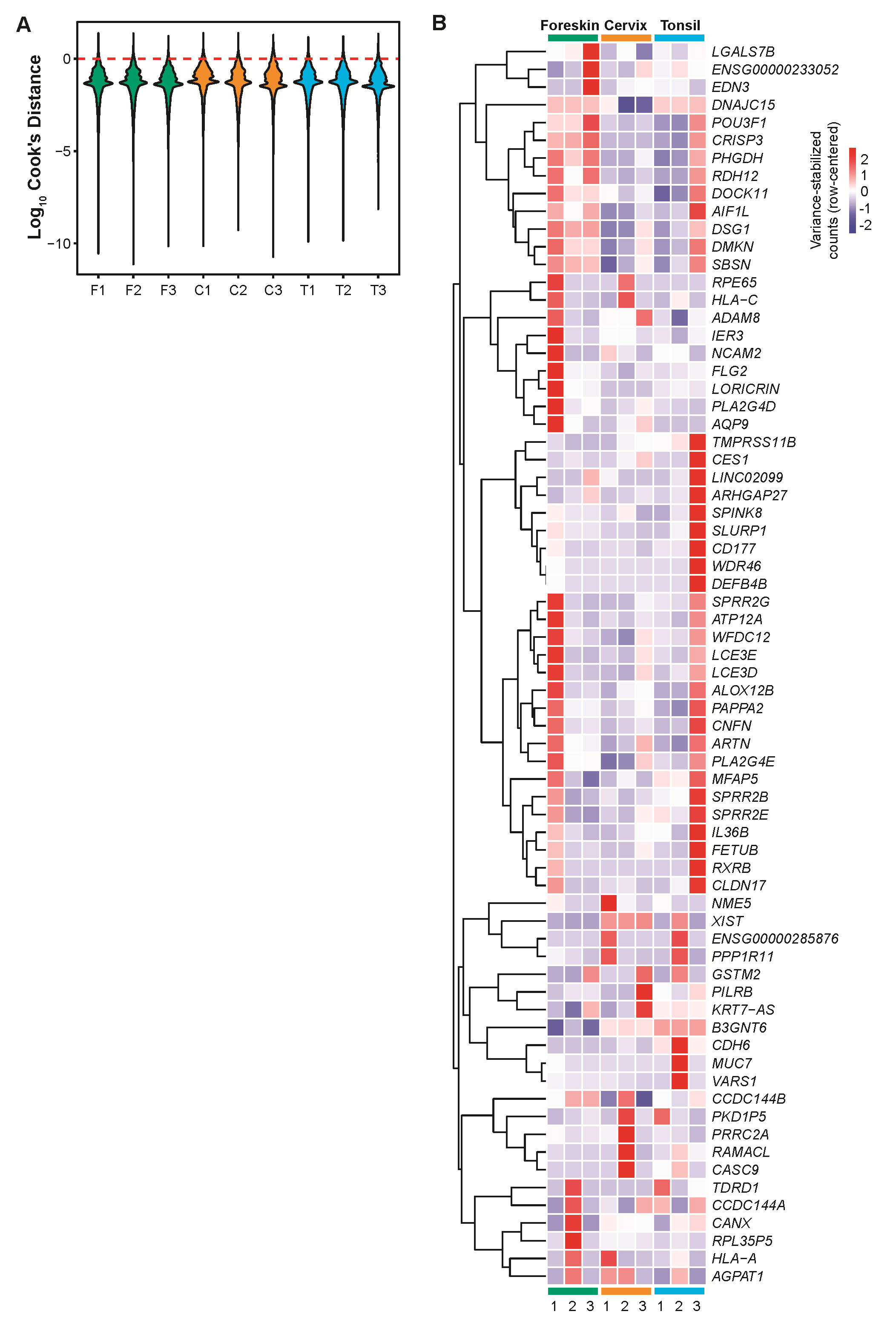

Supplement: S2 Fig — (A) Sample outlier quality control: violin plot of log10 Cook’s distances (y-axis) to assess if any individual samples (x-axis) were overtly influencing the DESeq2 analyses. All samples were approximately equal and we identified no sample outliers (i.e., violins were all below the red dashed line). (B) Heatmap of the 70 outlier genes identified by DESeq2. Scale bar represents row-centered variance-stabilized log2 fold change. (TIF) [file pone.0292368.s002.tif]

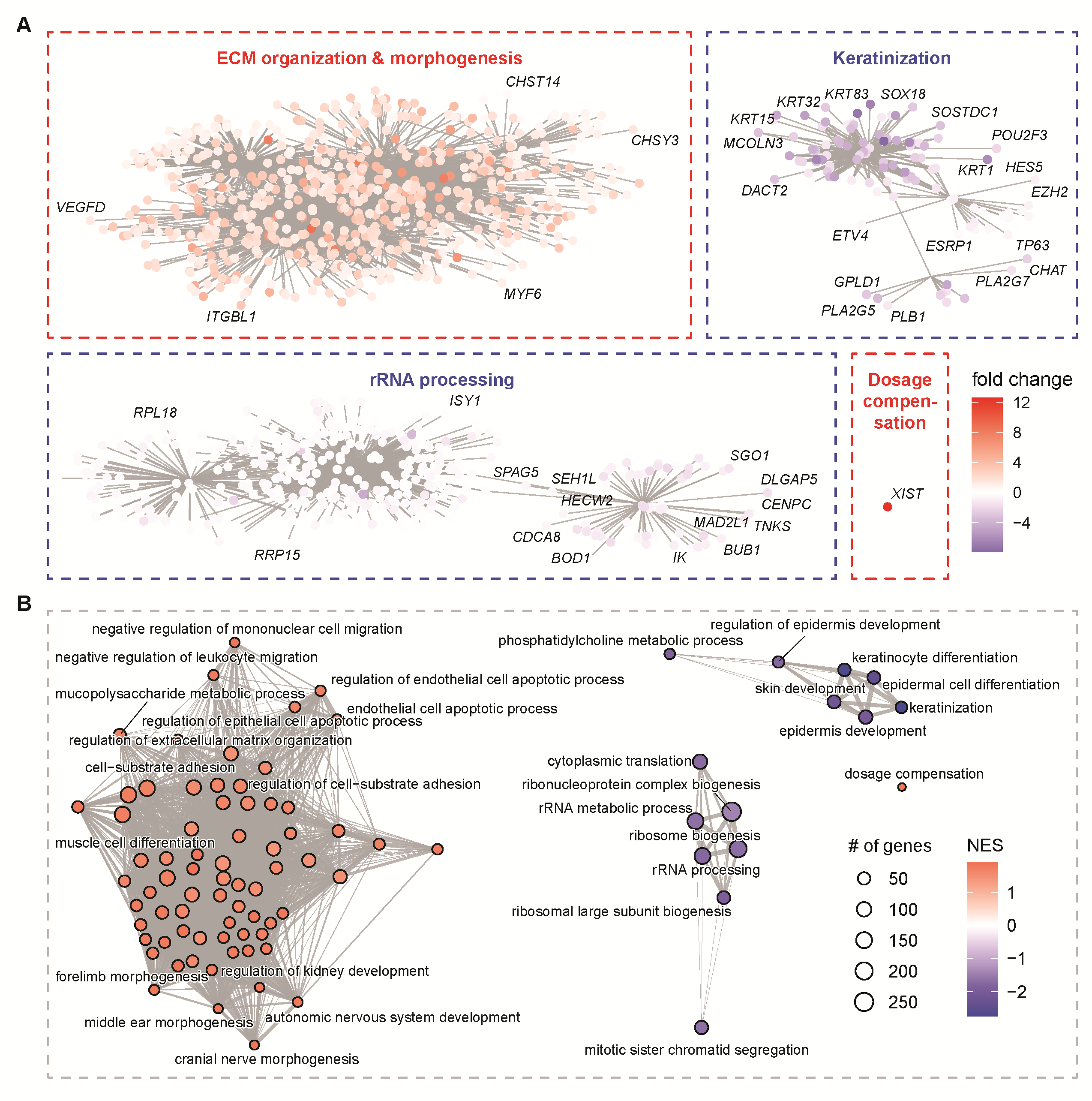

Supplement: S3 Fig — (A) Gene-concept network of the statistically significant Gene Ontology Biological Process (GO:BP) terms from Gene Set Enrichment Analysis (GSEA) of the pairwise contrast results. Nodes are the core enriched genes colored based on their log2 fold change (red = enriched and blue = depleted). Connected terms represent functional modules. (B) Enrichment map network of just the GO:BP terms colored based on normalized enrichment score (NES). (TIF) [file pone.0292368.s003.tif]

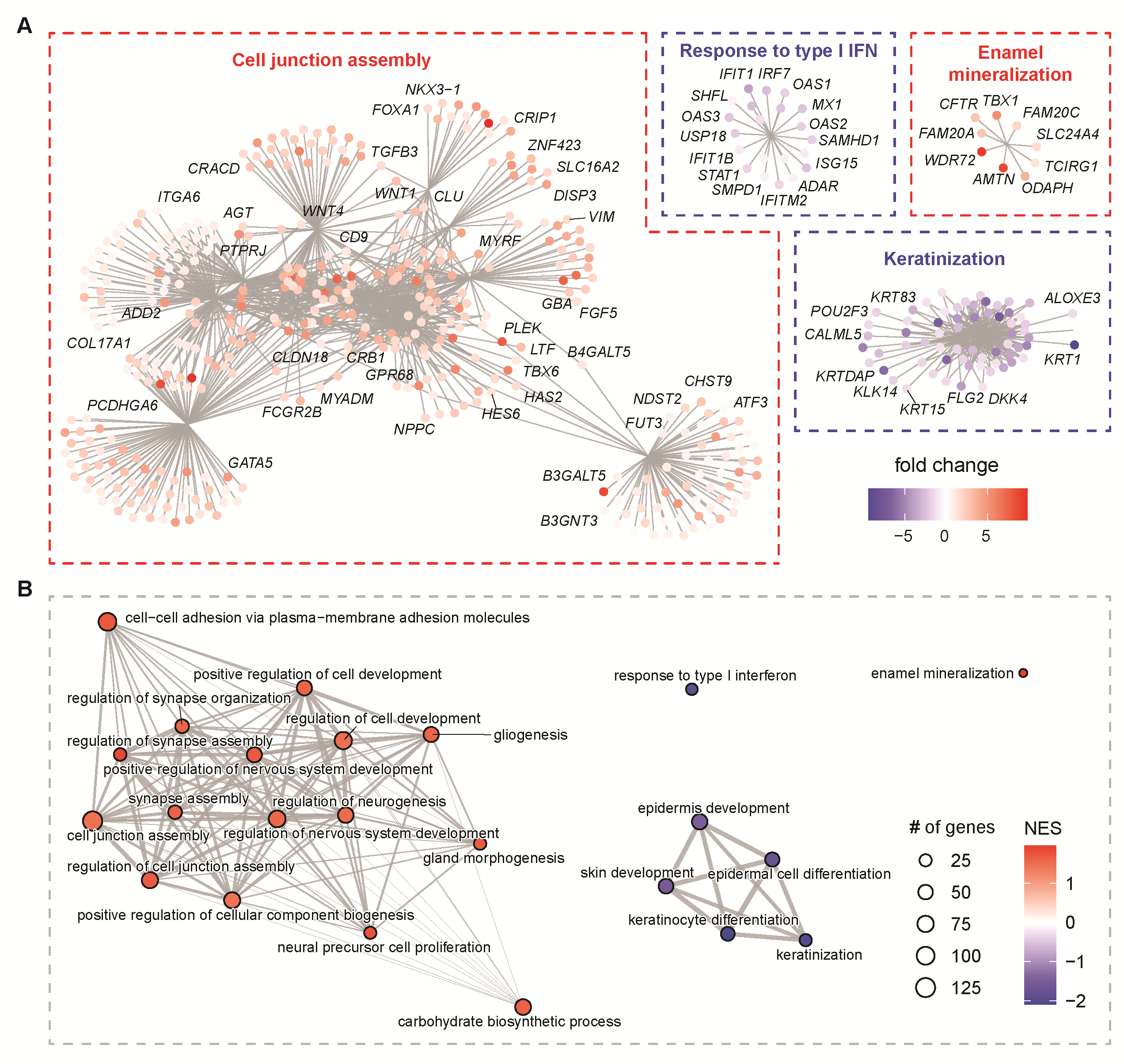

Supplement: S4 Fig — (A) Gene-concept network of the statistically significant Gene Ontology Biological Process (GO:BP) terms from Gene Set Enrichment Analysis (GSEA) of the pairwise contrast results. Nodes are the core enriched genes colored based on their log2 fold change (red = enriched and blue = depleted). Connected terms represent functional modules. (B) Enrichment map network of just the GO:BP terms colored based on normalized enrichment score (NES). (TIF) [file pone.0292368.s004.tif]

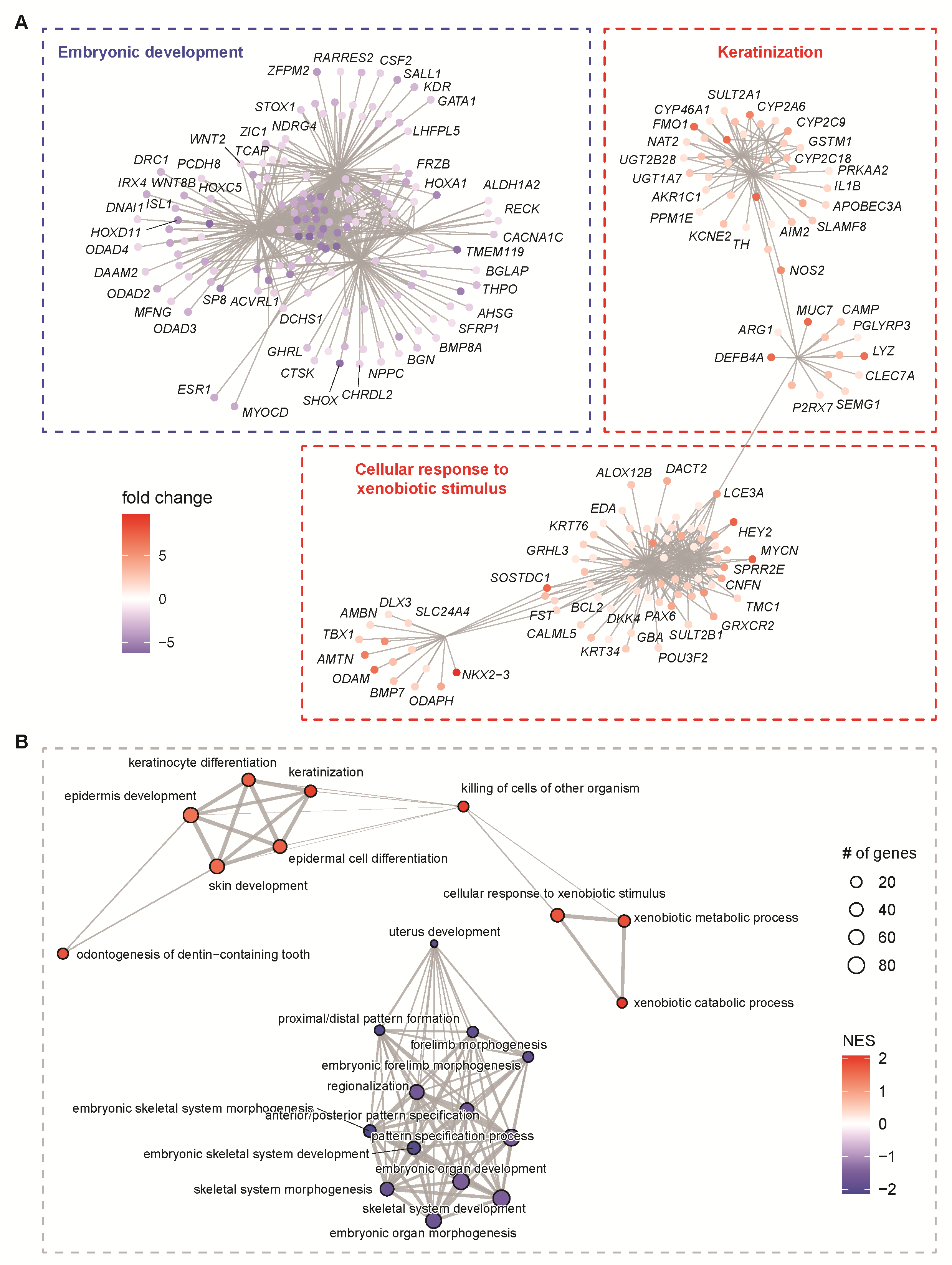

Supplement: S5 Fig — (A) Gene-concept network of the statistically significant Gene Ontology Biological Process (GO:BP) terms from Gene Set Enrichment Analysis (GSEA) of the pairwise contrast results. Nodes are the core enriched genes colored based on their log2 fold change (red = enriched and blue = depleted). Connected terms represent functional modules. (B) Enrichment map network of just the GO:BP terms colored based on normalized enrichment score (NES). (TIF) [file pone.0292368.s005.tif]

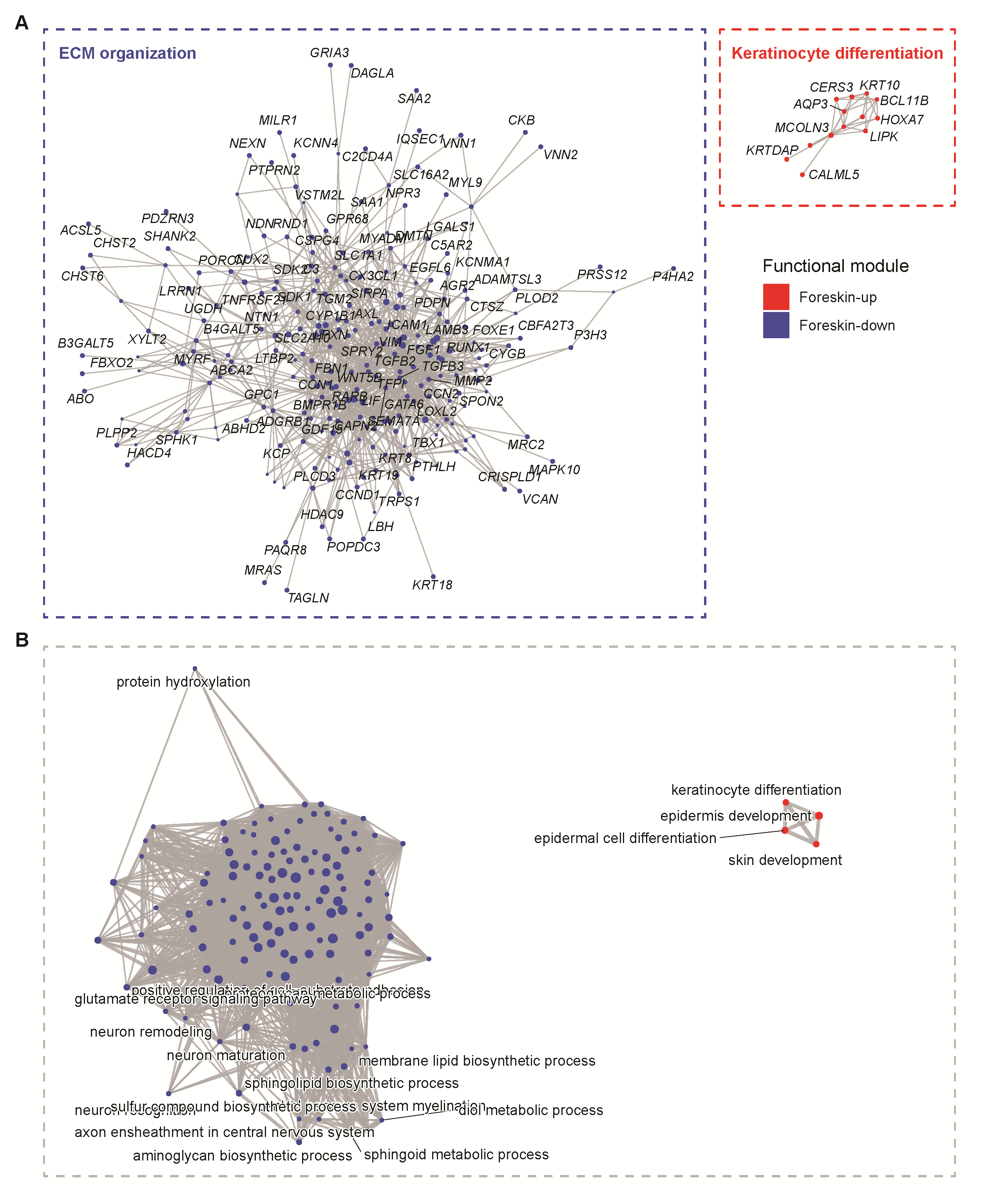

Supplement: S6 Fig — (A) Gene-concept network of the statistically significant Gene Ontology Biological Process (GO:BP) terms from over-representation analysis (ORA) of the tissue-specific differentially expressed genes. Term categories are the hub nodes, while the surrounding nodes are the core enriched genes. Colors: red = up, and blue = down). Connected terms represent functional modules. (B) Enrichment map network of just the GO:BP terms colored based on whether they are enriched (red) or depleted (blue). (TIF) [file pone.0292368.s006.tif]

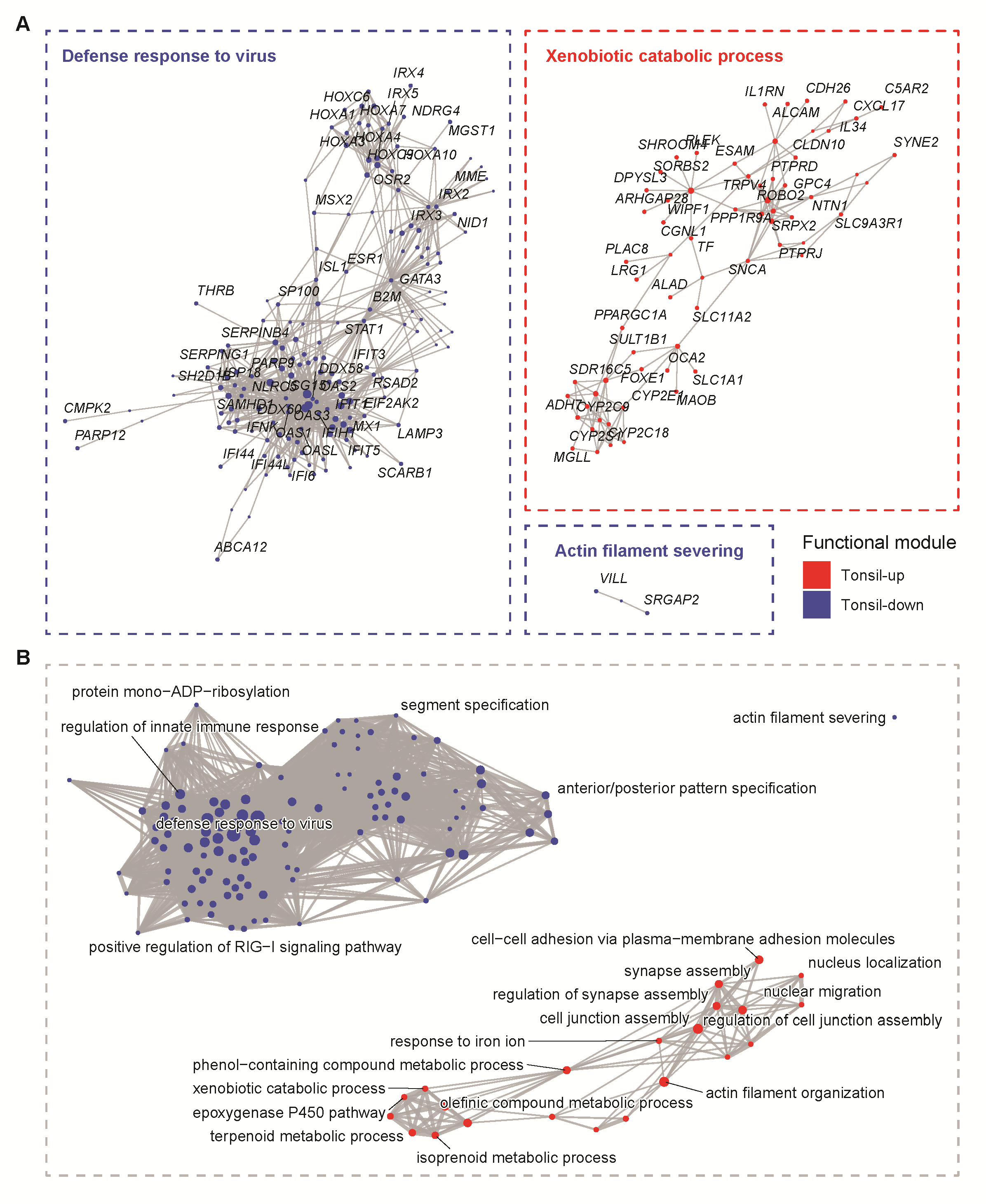

Supplement: S7 Fig — (A) Gene-concept network of the statistically significant Gene Ontology Biological Process (GO:BP) terms from over-representation analysis (ORA) of the tissue-specific differentially expressed genes. Term categories are the hub nodes, while the surrounding nodes are the core enriched genes. Colors: red = up, and blue = down). Connected terms represent functional modules. (B) Enrichment map network of just the GO:BP terms colored based on whether they are enriched (red) or depleted (blue). (TIF) [file pone.0292368.s007.tif]

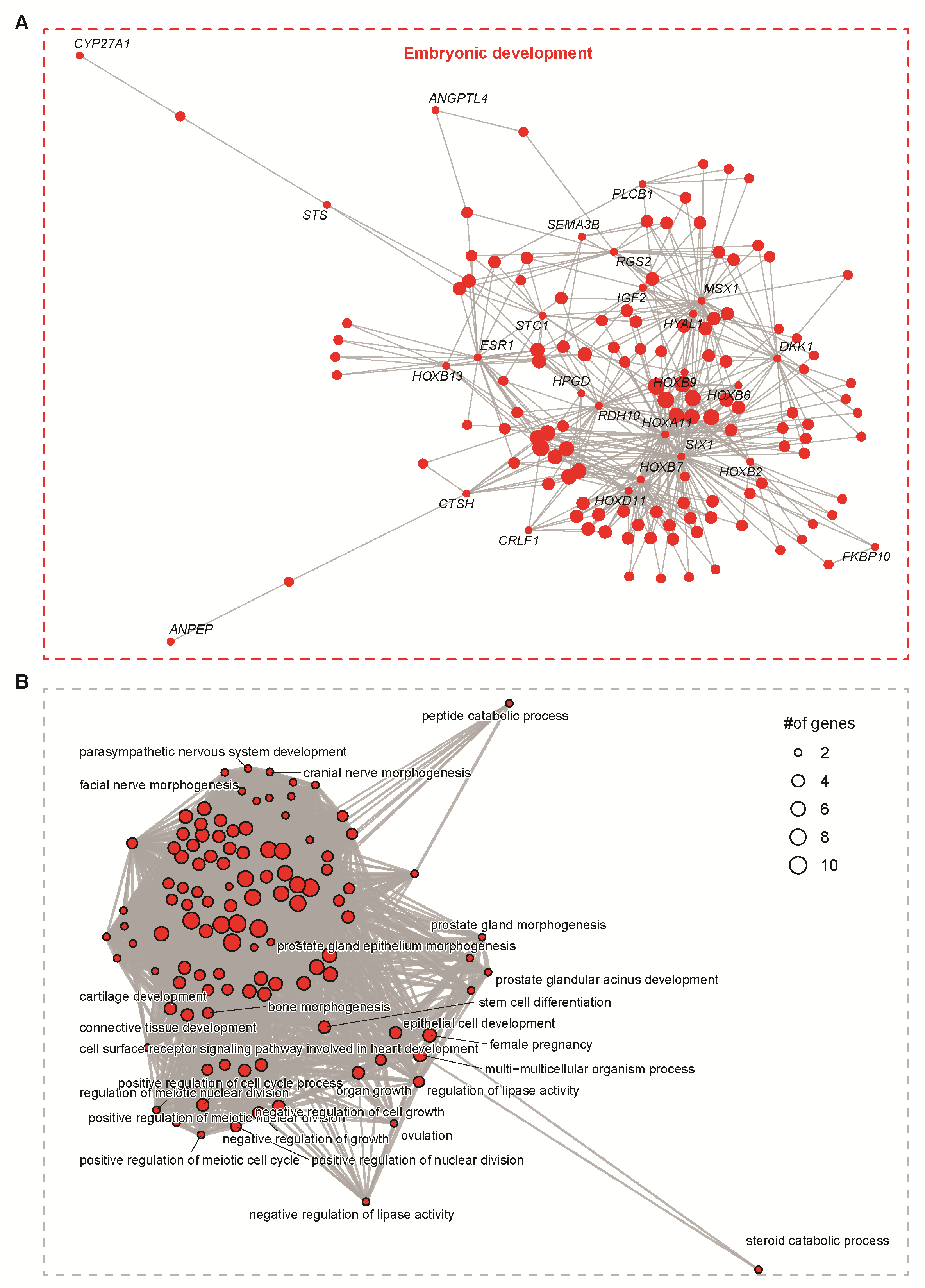

Supplement: S8 Fig — (A) Gene-concept network of the statistically significant Gene Ontology Biological Process (GO:BP) terms from over-representation analysis (ORA) of the tissue-specific differentially expressed genes. Term categories are the hub nodes, while the surrounding nodes are the core enriched genes. Colors: red = up). Connected terms represent functional modules. (B) Enrichment map network of just the GO:BP terms colored based on whether they are enriched (red). (TIF) [file pone.0292368.s008.tif]

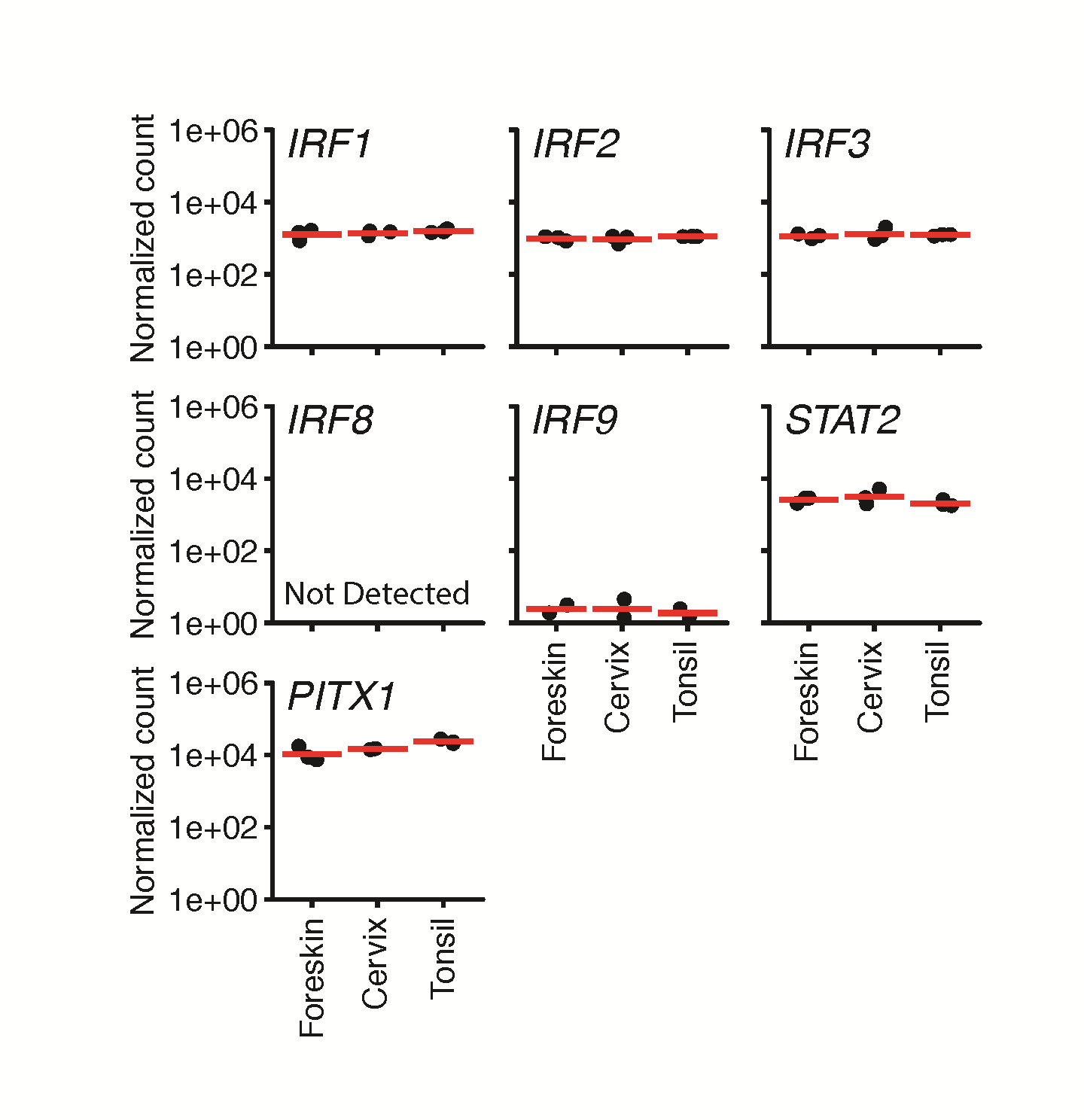

Supplement: S9 Fig — Log10-scaled normalized RNA-seq count data were plotted for genes encoding transcription factors (and complex components) identified via Integrated Motif Activity Response Analysis (ISMARA): IRF2_STAT2_IRF8_IRF1, IRF9, IRF3, and PITX1. These were binding motifs with a z-score larger than 2 whose target gene expression does not change between foreskin and cervix but are reduced in tonsil-derived tissue. (TIF) [file pone.0292368.s009.tif]
